# Supplementary material for: A Magnetic Reduced Graphene Oxide Nanocomposite: Synthesis, Characterization, and Application for High-Efficiency Detoxification of Aflatoxin B1
Source: Toxins (Basel). 2024 Jan 19;16(1):57. doi: 10.3390/toxins16010057 (PMC10818925; doi:10.3390/toxins16010057)
Supplement: Supplementary file 1 [file toxins-16-00057-s001.zip › toxins-2800928-supplementary.pdf]

toxins

MDPI

# Supplementary material: A Magnetic Reduced Graphene Oxide Nanocomposite: Synthesis, Characterization, and Application for High-Efficiency Detoxification of Aflatoxin $B_1$

**Table S1.** Nitrogen sorption-desorption experiments of the $Fe_3O_4$@rGOs produced under different preparation temperatures.

| Reaction temperature (°C) | Surface area ($m^2 \cdot g^{-1}$) | Pore volume ($cm^3 \cdot g^{-1}$) | Pore diameter (nm) |
|---|---|---|---|
| 180 | 80.240 | 0.063 | 3.066 |
| 200 | 87.094 | 0.483 | 3.428 |
| 220 | 57.145 | 0.351 | 3.070 |

**Table S2**. Maximum adsorption capacity (Qm/mg·$g^{-1}$) of $AFB_1$ by various absorbents in previous reports.

| Absorbents | Qm (mg·$g^{-1}$) | References |
|---|---|---|
| Functionalized nanoflower-like hydroxyl magnesium silicate | 27.34 | [19] |
| Modified nano-montmorillonite | 9.33 | [20] |
| Magnetic graphene composite adsorbents | 1.48 | [29] |
| Tannic acid pillared bentonite composite | 86 | [37] |
| Cross-linked chitosan | 5.67 | [41] |
| Grape pomace | 15.0 | [42] |
| Chitosan modified GO | 9.6 | [43] |
| Flower-like mesoporous magnesium silicate composites | 21.26 | [44] |
| Nonionic surfactant modifified montmorillonite | 2.78 | [45] |
| $Fe_3O_4$@ rGO | 82.64 | In this study |
